# Phase I trial evaluating safety and efficacy of intratumorally administered inflammatory allogeneic dendritic cells (ilixadencel) in advanced gastrointestinal stromal tumors

**DOI:** 10.1007/s00262-020-02625-5

**Published:** 2020-06-13

**Authors:** Robin Fröbom, Erik Berglund, David Berglund, Inga-Lena Nilsson, Jan Åhlén, Karin von Sivers, Christina Linder-Stragliotto, Peter Suenaert, Alex Karlsson-Parra, Robert Bränström

**Affiliations:** 1 Section of Endocrine and Sarcoma Surgery, Department of Molecular Medicine and Surgery, Karolinska Institutet, Stockholm, Sweden; 2 Department of Breast Cancer, Endocrine Tumors and Sarcoma, Division of Cancer, Karolinska University Hospital, Stockholm, Sweden; 3 Department of Transplantation Surgery, Department of Clinical Science, Intervention and Technology, Karolinska Institutet, Stockholm, Sweden; 4 Department of Transplantation Surgery, Karolinska University Hospital, Stockholm, Sweden; 5 Department of Immunology, Genetics and Pathology, Section of Clinical Immunology, Uppsala University, Uppsala, Sweden; 6 Department of Radiology, Karolinska University Hospital, Stockholm, Sweden; 7 Immunicum AB, Stockholm, Sweden

**Keywords:** Gastrointestinal stromal tumor, Immunotherapy, Cell therapy, Ilixadencel, Tyrosine kinase inhibitor, Dendritic cells

## Abstract

**Background:**

The majority of patients with advanced gastrointestinal stromal tumor (GIST) develop resistance to imatinib, and subsequent treatments have limited efficacy. Ilixadencel (allogeneic inflammatory dendritic cells) is a cell-based immune primer injected intratumorally that previously has been clinically investigated in metastatic renal cell carcinoma and hepatocellular carcinoma.

**Methods:**

The trial was a single arm phase I trial assessing safety and efficacy of ilixadencel in subjects with progressing advanced/metastatic GIST despite ongoing treatment with second or later lines of tyrosine kinase inhibitors (TKI). Three patients were progressing while on sunitinib (second line), one on regorafenib (third line), and two on pazopanib (fourth line). TKI treatment was maintained throughout, while two intratumoral injections of ilixadencel (10 × 10^6^ viable and HLA-DR expressing cells per dose) were administered.

**Results:**

No severe adverse events were found to be related to ilixadencel administration. Four patients showed continued tumor progression at 3 months per RECIST 1.1 and Choi criteria. One patient (on third line regorafenib) had stable disease for 9 months and another patient (on second line sunitinib) had stable disease at end of study (12 months) as per RECIST 1.1. These two patients developed a partial response as per Choi criteria with a duration of 3 and 6 months, respectively. The median progression-free survival (PFS) was 4.0 months.

**Conclusion:**

Ilixadencel treatment presented an acceptable safety profile among advanced GIST patients who developed resistance to TKI. Encouraging radiological tumor responses were detected in 33% of treated patients, supporting further investigation.

**Clinical trial registration** www.clinicaltrials.gov; NCT: 02432846; registration date: February 22, 2016.

**Electronic supplementary material:**

The online version of this article (10.1007/s00262-020-02625-5) contains supplementary material, which is available to authorized users.

## Introduction

Gastrointestinal stromal tumor (GIST) is the most common sarcoma of the gastrointestinal tract, most frequently located in the stomach or small intestine. About 85–90% of GIST possess a *gain*-*of*-*function* mutation in the *KIT* or *PDGFRA* gene [1]. Two decades ago, the tyrosine kinase inhibitor (TKI) imatinib was introduced in the treatment of GIST and thereby constituted the first approved targeted therapy for solid tumors [2]. Since then, the TKIs sunitinib and regorafenib have been approved for treatment of imatinib (or sunitinib as second-line) refractory disease [3, 4]. Despite this, resistance and disease progression ultimately affect the majority of patients, and GIST is inherently insensitive to chemotherapy, with historical data reporting response rates using chemotherapy from 0 to 15% [5]. Durable remissions are uncommon due to the resistance development and few alternative treatments are available beyond TKIs.

Further to its direct antitumoral activities, TKIs have been shown to possess immunomodulatory properties. Imatinib potentiates antitumor T-cell response in GIST by activating CD8^+^ T cells (CTLs) and induce apoptosis of regulatory T cells (T_regs_) through down-regulation of indoleamine 2,3-dioxygenase (Ido) [6]. Combining imatinib with CTLA-4 blockade further enhanced the antitumoral effect in GIST [6]. PD-1 and PD-L1 blockade as single treatment showed no antitumoral activity in vivo, while efficacy improved when it was combined with imatinib, by down-regulating IFN-γ-related genes and suppressing PD-L1 expression on tumor cells [7]. Imatinib has also been shown to polarize tumor-associated macrophages (TAM) in GIST from M1 to the immunosuppressive M2 TAM, upon imatinib resistance development, M2 TAM polarizes back to M1 TAM [8]. NK cells become activated by imatinib indirectly, through increased cross-talk between dendritic cell (DC) and NK cells, that is KIT-inhibition dependent, leading to increased levels of NK cell activation and IFN-γ production [9]. Sunitinib has been shown to create a less immunosuppressive microenvironment in renal cancer by decreasing myeloid-derived suppressor cells (MDSC), which are known to inhibit T-cell sensitization to tumor antigens and T_regs_ [10, 11]. The reduced number of MDSCs is not correlated to the antitumoral effect of sunitinib [11]. In GIST, the intratumoral immunological landscapes have been reported in several studies [12–14]. The most common immune cells are TAMs, followed by CD3^+^ lymphocytes, and other cells are more sparse. The ratio between CD8^+^ lymphocytes and T_regs_ was found to be low, and most TAMs were M2-polarized, collectively interpreted as skewed towards immune suppressive profile [14]. Immune cells can predict progression-free survival (PFS) in GIST, where NK cells and CD3^+^ T cells, but not T_regs_, being independently correlated to PFS [13]. The immune infiltrate profiles also vary depending on mutational status (*KIT* vs *PDGFRA* mutations) [15]. Clinically, immune checkpoint blockade combining CTLA-4 with tyrosine kinase inhibitor showed no clinical benefit in GIST [16].

Ilixadencel is a monocyte-derived allogeneic DCs’ product stimulated with a combination of potent activators. In a mouse model, administration of activated allogeneic mouse DCs intratumorally induced NK- and T-cell recruitment [17, 18]. Similarly, the proposed mechanism of action by ilixadencel [19] following injection is by secreting chemokines that recruit immune cells (NK cells, pre-DCs, and T cells) into the tumor. Upon interaction with the allogeneic activated DC cells, NK cells are activated and mediate tumor cell elimination resulting in tumor antigen release. A combination of factors from NK cell (IFN_-_γ) and ilixadencel DCs (TNF-α/IL-1β) will lead to increased cross-presentation as well as maturation of endogenous DCs. DCs with antigen captured will migrate to the lymph node and activate tumor-specific T cells. Clinically, two phase I/II trials have evaluated ilixadencel’s safety and efficacy, in metastatic renal cell carcinoma [20] and hepatocellular carcinoma [21], and have demonstrated favorable safety profiles. Recently, a randomized phase II trial (clinicaltrials.gov NCT: 02432846) has been completed and final results are awaited.

Provided the limited alternative treatments to TKI in GIST, and the likely immune suppressive environment within GIST tumors, we hypothesized that activated allogeneic dendritic cells could possibly—by acting as an immune primer—hold the potential to prime a T-cell-mediated antitumor response. Combining with TKI treatment might also attenuate the immune-stimulative effect. Therefore, we wanted to explore the safety of using ilixadencel as an immune primer to overcome intratumoral tolerance.

## Materials and methods

### Trial design and clinical setting

**Fig. 1:**
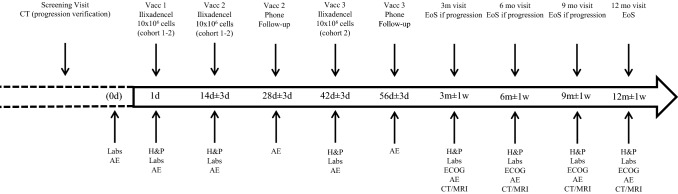
Schematic overview of the study design.* AE* adverse event,* CT* computed tomography,* d* days,* ECOG* Eastern Cooperative Oncology Group,* EoS* end of trial,* H&P* history and physical examination,* Labs* laboratory tests,* m* months,* MRI* magnetic resonance imaging,* Vacc* vaccination

The trial (registered at clinicaltrials.gov: NCT02689644) was designed as a prospective, single-armed, open-label phase 1 study to assess safety and efficacy of ilixadencel injection in subjects with advanced unresectable and/or metastatic progressing GIST despite ongoing treatment with second or later line of TKI treatment. The initial trial (Fig. 1) was designed to consist of two cohorts (modified 3 + 3 design per cohort) and 12 patients in total, with one arm receiving a dose-escalated schedule. Following inclusion of six patients, the trial was closed due to slow subject recruitment. All included patients followed the arm with a non-dose-escalated schedule.

The trial was conducted at the Department of Endocrine and Sarcoma Surgery, Karolinska University Hospital, Stockholm, Sweden. It was approved by the institutional review board (Dnr 2015/1619-31), as well as the Swedish Medical Products Agency (Dnr 5.1-2015/77670). All included patients signed an informed consent, in line with Declaration of Helsinki, prior to study participation. The data safety monitoring board (DSMB) included a minimum of two independent physicians with relevant expertise in oncology and clinical research at all times. Based on safety information, the DSMB directed recommendations to the Sponsor concerning continuation, modification, and trial termination.

### Patient eligibility

Men and women, at least 18 years of age, with a diagnosis of unresectable or metastatic GIST that had progressed despite second-, third-, or fourth-line treatment with a TKI were considered. The size of the lesion had to be of at least 3 cm in longest unidimensional diameter measured by computed tomography (CT). Patients were excluded if performance status according to Eastern Cooperative Oncology Group (ECOG) was > 2, abnormal hematological parameters. Patients with viral disease (hepatitis B, C, and HIV), active autoimmune disease which required immunosuppressive, or with previously major reaction/adverse event (AE) in connection with previously made vaccinations or transfusion of blood products were excluded from study inclusion. For detailed inclusion and exclusion criteria.

### Preparation of ilixadencel

Ilixadencel was prepared from healthy blood donors, in which donor screening and donor eligibility are regulated by country-specific law and implemented EU directives. Cells in the leukapheresis and thereafter fractionated by elutriation in a close system, ELUTRA^®^ (Gambro BCT). The elutriation results in a cell product in fraction 5 that contains > 90% CD14^+^ monocytes. These cells are used for differentiation (using the well-established differentiation cocktail GM-CSF plus IL-4) and activation (R848.poly-IC and IFN-gamma) into proinflammatory DCs. The final drug product is cryopreserved cells formulated in human plasma and 10% DMSO [19]. The requirements of the product post-thawing were a cellular viability of > 70%, HLA-DR expression of > 50%. Furthermore, it was required to produce > 7500 pg RANTES/mL/10^6^ cells.

The ilixadencel batch used for the first four GIST patients was produced at Cancer Center Karolinska, Karolinska University Hospital, Sweden. Immediately before administration to the patient, the frozen vials were thawed and the cells were washed and resuspended in 0.15 M saline with 2% human serum albumin before administration. The last two patients received ilixadencel from a batch produced at BioNTech, Idar-Oberstein, Germany (after a standard tech transfer). These cells were used as a direct-injectable product after thawing without any additional preparation steps prior to the intratumoral administration.

### Treatment

The dosing regimen was chosen from a previous first in-human trial in metastatic renal cell carcinoma patients [20] where doses of 5 × 10^6^ (low dose), 10 × 10^6^ (medium dose), and 20 × 10^6^ (high dose) were used. A combination of safety, immunological, and efficacy parameters were considered when selecting the medium dose of 10 × 10^6^ cells for this trial.

The first dose of ilixadencel dose containing 10 × 10^6^ viable HLA-DR^+^ cells was injected on study day 1, and the second dose on day 14 ± 3 days. The starting dose could be reduced to 5 × 10^6^ cells for subjects where limiting toxicities (LTs) were observed. The injections were ultrasound-guided; all injections were done at the Department of Radiology at Karolinska University Hospital by experienced radiologists. The target lesions were decided at the discretion of the radiologist. Practically, the injected lesions were also evaluated for size, localization, and viable tumor tissue (i.e., intratumoral vasculature). After injection, patients were observed for at least 6 h for possible adverse events following injection of ilixadencel. The patients continued TKI treatment after ilixadencel administration. If progression occurred until the 3 month screening visit, the subject performed an End of Study visit. If stable disease, the subject continued with TKI and follow-up until progression of disease, and controls as outlined in the protocol.

### Primary and secondary objectives

The primary objectives were to evaluate ilixadencel’s safety profile and identify LTs, if any. Secondary objectives were to evaluate tumor response by CT, evaluate progression-free survival, changes in ECOG score, and to evaluate potential auto- and alloimmunization. Blood samples for immuno-monitor analysis were obtained at screening, at baseline and at 3-month post-vaccination visit.

### Safety and toxicity

Adverse events (AEs) were monitored throughout the study and graded according to the National Cancer Institute (NCI) common toxicity criteria (CTCAE) version 4.03. At each trial visit in the clinic, vital signs, physical examination, as well as safety lab were collected. Before each injection, additional blood samples were drawn for hemoglobin, white blood cell count, platelets, and coagulation status. Vital signs were taken at all trial visits. After injection, more thorough monitoring was employed for 6 h.

To evaluate potential treatment-induced alloimmunization at the humoral level, and possible autoimmunization, blood samples were collected at baseline and 3-month clinical follow-up. The detection of donor (vaccine cell)-specific alloantibodies was analyzed with cytometry-based (Luminex) technique. Serum samples for this assay were collected twice, before the first vaccination and at the 3-month follow-up visit. If alloantibodies specific for MHC class I or class II antigens on the vaccine cells were not present before vaccination but were present in the 3-month follow-up, the results were considered as vaccine-induced. For autoimmunization, screening of the following nuclear antigens was performed: SSA (Ro52 and Ro60), SSB, Sm, RNP68, Scl-70, centromeres, and Jo-1 in serum.

### Evaluation of tumor response

Tumor response was evaluated by CT (also MRI was acceptable modality, if MRI was chosen, it was the preferred modality for follow-up scans) at baseline and thereafter at 3-month intervals, if no progression occurred, after the first dose of ilixadencel until 12 months after first vaccination. The baseline imaging was undertaken within 28 days before the first injection, and was considered as the baseline measure in the trial. The tumor stage was classified as progressive disease (PD), stable disease (SD), partial response (PR), or complete response (CR) according to modified response evaluation criteria in solid tumors RECIST 1.1 and Choi criteria. All CT-evaluated lesions consisted of one injected lesion and one non-injected lesion. In MRI evaluation (one patient), three lesions were used, one injected lesion and two non-injected lesions.

### Statistical analysis

All endpoints are evaluated by descriptive methods.

## Results

### Patient characteristics and treatment

Between June 2016 and May 2018, seven patients were screened for study participation; six patients were enrolled and received two doses of intratumorally injected ilixadencel at a dose of 10 × 10^6^ cells. One patient was excluded after screening due to abnormal hematological parameters. One patient had an abnormal hemoglobin value on screening (hemoglobin < 100 g/L), though included upon investigators discretion.

The patients had a mean age of 57 (46–82), three males (50%), ECOG score varied between participants with ECOG 0 (3 patients), ECOG 1 (1 patient), ECOG 2 (2 patients). Prior to ilixadencel administration, all patients had received TKI treatment with two agents (50%), three (16.7%), or four (33.3%) previous agents (Table 1). Three of the patients had progressed on sunitinib (second-line), two on pazopanib (fourth-line), and one while on regorafenib (third-line). Four patients had metastases in the liver or in the abdominal cavity; the remaining two had unresectable disease (Table 2). All injections were performed in lesions located in the abdomen and were successfully performed ultrasound-guided.

**Table 1:** Patient characteristics

Ilixadencel treatment line	Number of patients (%)	Dose ilixadencel
Second line	3 (50%)	10 × 10^6^ twice
Third line	1 (17%)	10 × 10^6^ twice
Fourth line	2 (33%)	10 × 10^6^ twice

* M* male,* F* female,* ND* not determined

**Table 2:** Ilixadencel treatment line including doses of ilixadencel for each participant in each group

Patient	Years (age)	Sex	Primary tumor site	Mutation in primary tumor	Site of disease	ECOG
1	46	M	Small intestine	*KIT* Exon 9	Abdominal cavity	0
3	59	M	Small intestine	*KIT* Exon 9	Abdominal cavity	2
4	56	F	ND	No mutation in *KIT or PDGFRA*	Abdominal cavity, liver, cutaneous	0
5	51	F	Small intestine	*KIT* Exon 9	Peritoneal, liver, lymph	1
6	50	M	ND	*KIT* Exon 11	Abdominal cavity	0
7	82	F	Small intestine	*KIT* Exon 11	Abdominal cavity, liver	1

* M* male,* F* female,* ND* not determined

### Treatment safety

During the trial, five out of six patients experienced a total of 19 AEs. Grade 1 was the most common with 10 AE reported in 4 patients (66.7% of total); 8 grade 2 in 2 patients (33.3%), and 1 patient with grade 3 (16.7%). The adverse events related to ilixadencel, as defined by probable or possibly causative of ilixadencel, were 6 AEs shown in Table 3. The AEs related to ilixadencel treatment was fever and chills (50%), abdominal pain (33%), and discomfort at injection site (17%). The grade 3 AE was related to general health deterioration and not related to ilixadencel treatment. No clinically relevant abnormalities were noted post-treatment with regard to hematology, biochemistry, coagulation, or serology. All ilixadencel-related AEs were transient in nature and had resolved before the end of the study. The radiologist injecting ilixadencel reported no immediate injection complications. No dose reduction was necessary.

**Table 3:** Adverse events related to ilixadencel, interpreted as probable or possible causative

Reported adverse events	Frequency (% of total AE)
Fever/influenza-like symptoms	3 (50%)
Abdominal pain	2 (33%)
Administration site discomfort	1 (17%)

### Tumor response

**Fig. 2:**
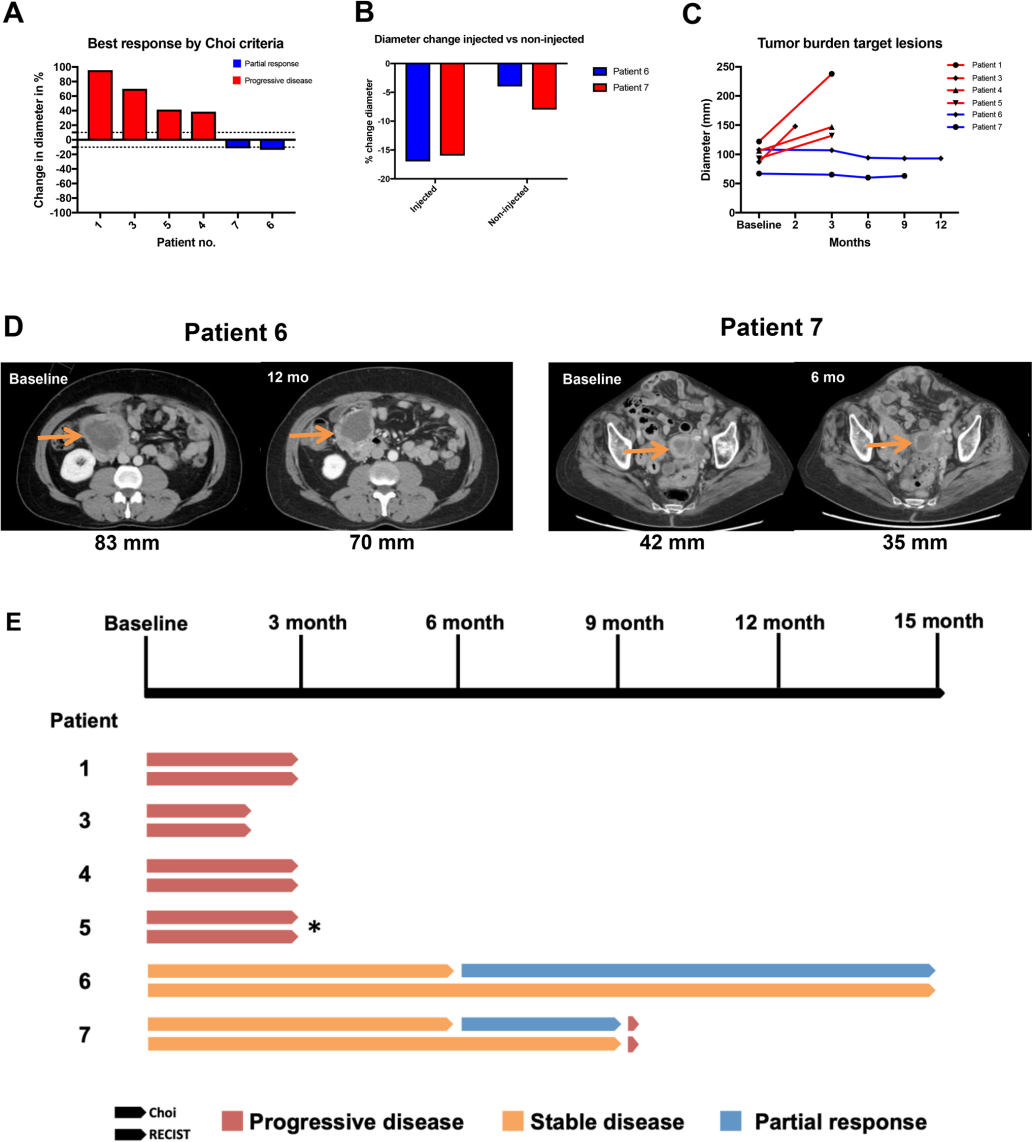
Tumor responses after ilixadencel treatment. **a** Waterfall plot showing best response according to Choi criteria, expressed as percentage change from baseline. Dotted lines marks ±10% used as cutoff for tumor diameter, patient 3 CT scan was 2 month from baseline; **b** Indicating the diameter change of injected lesion and non-injected lesion in patient with partial response (patient 6 and 7); **c** Spider plot indicating tumor burden (in mm) of target lesions at different time points. **d** CT scans of best responses, indicating dimension (mm) at baseline and at best follow-up. **e** Swimmer plot of participants and responses at indicated follow up CT scans, for Choi and RECIST v1.1, respectively. Patient 3 were assessed at 2 months. * Denotes MRI evaluation, where diameter measurement was performed

Radiological measurements at indicated follow-up CT/MRI scans were reviewed by one senior sarcoma radiologist of the injected lesion and also non-injected tumor lesion. MRI was performed of patient no 5, in which longest unidimensional diameter was used for single parameter evaluation. Three patients showed tumor progression at 3 months per RECIST 1.1 and Choi criteria, and one patient (patient no 3) showed clinical deterioration and CT scan was performed after 2 months, which showed progressive disease. One patient (on third-line regorafenib) had stable disease for 9 months and another patient (on second line sunitinib) had stable disease at end of study (12 months) as per RECIST 1,1. These two patients developed a partial response as per Choi criteria with a duration of 3 and 6 months, respectively (Fig. 2). In patient no 6, the corresponding best response in diameter was − 16% and − 8% in injected and non-injected lesion, respectively. In patient no 7, the diameter change was − 17% and − 4% respectively. For all patients with progressive disease, the non-injected lesion increased in size.

The median PFS for the trial cohort was 4.0 months (95%; CI 3.2–4.8); the median OS was 19.0 months (95% CI 11.8–26.2). One patient displayed a change in ECOG score, increasing from 2 at the screening to 3 at the last time point the patient was assessed, whereas all other patients had maintained their ECOG scores throughout the study. Disease progression was seen in five patients: four at 3 months, one at 9 months, and one patient showed stable disease at study end (12 months), Fig. 2E.

### Development of allo- and autoimmunization

Three subjects (50%) had developed donor-specific antibodies, indicating alloimmunization. There were no detectable nuclear antibodies neither at screening nor at 3-month clinical follow-up.

## Discussion

This is the first trial evaluating the safety and efficacy of ilixadencel in advanced GIST when combined with second or later lines of TKI treatment despite ongoing tumor progression. Performing intratumoral injections of ilixadencel at 10 × 10^6^ cells/dose proved feasible, with an acceptable safety profile and with two out of six patients displaying stable disease for 9 months or longer as per RECIST 1.1 criteria and partial tumor regression as per Choi criteria with a duration of 3 and 9 months, respectively. All patients continued with the TKI they were progressing on; therefore, the observed possible effects were likely attributed to ilixadencel treatment and not to change of TKI.

The most commonly reported AEs were fever, which is likely attributed to the proinflammatory DCs that induce an inflammatory response. There were no clinical or laboratory signs of autoimmunity. The safety profile seen in the present trial is in concordance with the previous trials with ilixadencel [20, 21], and supports that cell therapy using ilixadencel is safe in advanced GIST patients. Comparing the safety profile of ilixadencel with tyrosine kinase inhibitor treatment in naïve patients, the latter has reports about 40% grade 3 or higher in imatinib 400 mg/daily, which increases to 63% in high dose 800 mg/daily [22], and for regorafenib approximately 20% grade 3 or higher [4]. Taken together, ilixadencel is a safe alternative with limited toxicities, even when combined with TKIs. The development of alloantibodies (i.e., vaccine-specific) occurred in 50% of patients, which could be compared to previous studies of ilixadencel where 25% and 64%, in mRCC and HCC, respectively, developed alloantibodies [20, 21], indicative of a humoral immune response. However, this alloimmunization was not seen in patients with partial response.

In GIST, Choi criteria are more accurate compared to RECIST criteria, for evaluating imatinib response, mostly because imatinib is considered to be more cytostatic rather than cytotoxic [23, 24]. The Choi criteria also take into account the density (measured by Hounsfield unit [HU]), in addition to the longest diameter. Little is known about the most optimal way to evaluate responses to immune primer treatment. One patient (on third-line regorafenib) had stable disease for 9 months and another patient (on second-line sunitinib) had stable disease at end of study (12 months) as per RECIST 1.1, clinical follow-up outside the study at 15-month post-injection. These two patients developed a partial response as per Choi criteria with a duration of 3 and 9 months, respectively. The non-injected lesions decreased comparatively less (4–8% vs 17–18%) than injected. Even though small sample size, this could suggest a tumor-specific response rather than a non-specific local inflammatory response within the injected tumor. In the non-responders, no decrease of lesion size occurred in any lesion. Moreover, the proportion of GIST patients with stable disease for 9 months or longer during treatment with sunitinib or regorafenib is below 30% [3, 4]. The tumor response as per Choi criteria varies between the different TKIs; for imatinib, the overall response rate (ORR) was 53.7% [25]; for sunitinib used as a second-line treatment, ORR has been reported to be around 7–13% depending on dosing regimen [3, 26]; and ORR for regorafenib as third line was 4.5% [4].

Interestingly, both patients that displayed partial response had a *KIT* exon 11 mutations in their primary tumor, in contrast to the other patients (Table 1). *KIT* exon 11 mutation is the most common mutation in GIST [27]. By identifying the driver mutation, with mapping of possible neoepitopes (8–10 mer epitopes) with subsequent binding affinity testing against HLA molecules, it was shown that D842V *PDGFRA*-mutated produce a larger amount of neoepitopes compared to *KIT* or other *PDGFRA*-mutated GISTs, and *PDGFRA-*mutated GISTs were indeed shown to contain an increased amount of cytolytic immune cells [15]. Additionally, GIST is a tumor with relatively low mutational burden in comparison to tumors where successful outcomes from immunotherapy have been observed [28]. The present trial suggests that immunotherapeutic approaches are a viable option in low mutational burden tumors, as well. Moreover, in contrast to the four first enrolled patients, these two responding patients with *KIT* exon 11 mutation received the direct-injectable ilixadencel product. Without the access to pre- and post-treatment tumor biopsies or peripheral blood immune cell samples in the present study, the immune priming effect of ilixadencel, including potential activation of tumor-specific T cells, was, however, not able to be assessed.

Although the study includes a small number of patients, ilixadencel appears to possess a high safety profile upon injection in patients with advanced GIST already on TKI therapy, which further adds to the favorable safety profile seen upon administration in other tumors [20, 21]. The promising tumor regression response seen in two GIST patients supports further investigation and optimization with regard to ilixadencel dosing level and number of doses.

### Electronic supplementary material

Below is the link to the electronic supplementary material.

Supplementary file 1 (docx 16 kb)
