# Supplementary material for: Phase I trial evaluating safety and efficacy of intratumorally administered inflammatory allogeneic dendritic cells (ilixadencel) in advanced gastrointestinal stromal tumors
Source: Cancer Immunol Immunother. 2020 Jun 13;69(11):2393–401. doi: 10.1007/s00262-020-02625-5 (PMC7568699; doi:10.1007/s00262-020-02625-5)
Supplement: Supplementary file 1 — (docx 16 kb) [file 262_2020_2625_MOESM1_ESM.docx]

**SUPPLEMENTAL INFORMATION/DATA**

1. **Supplemental information 1.** Detailed inclusion and exclusion criteria used in the study.

**Diagnosis and main eligibility criteria:**

**Diagnosis:**

Gastrointestinal stromal tumor (GIST)

**Inclusion criteria:**

- Adults (≥18 years) who have provided written informed consent
- Diagnosis of GIST (according to modified NIH criteria, 2011), where curative excision is no longer an option, i.e. confirmed unresectable/metastatic GIST, and that has progressed on second, third, or fourth line TKI treatment
- Radiologically measurable tumor(s), i.e. ≥3 cm in longest uni-dimensional diameter (measured by CT)
- Clinical and/or CT verified disease progression despite second, third, or fourth line treatment with a TKI
- Females: post-menopausal for more than one (1) year or, if of childbearing potential, using a highly efficient method of contraception (i.e. a method with less than 1% failure rate [e.g. sterilization, hormone implants, hormone injections, some intrauterine devices, or vasectomized partner with combined use of condom and/or birth control pills]) during trial participation. Female of childbearing potential must have a negative blood pregnancy test at Screening and a negative blood/urine test within one (1) day before each Intuvax dose
- Males: agreeing to use condoms during the trial participation or male having a female partner who is using a highly efficient method of contraception (see above) during the partner’s trial participation

**Exclusion criteria:**

- Performance status ECOG >2
- Known major reaction/adverse event (AE) in connection with previously made vaccination (e.g. asthma, anaphylaxis or other serious reaction) or with previous transfusions of blood products
- Autoimmune disease requiring treatment with systemic immunosuppressive agents e.g. inflammatory bowel disease, multiple sclerosis, sarcoidosis, psoriasis, autoimmune hemolytic anemia, rheumatoid arthritis, SLE, vasculitis, Sjögren's syndrome, scleroderma, autoimmune hepatitis, and other rheumatological diseases.
- Tested positive for HIV
- Active virus disease (hepatitis B or C
- Ongoing infection that requires parenteral antibiotics/antiviral medication
- Oral corticosteroids exceeding 10 mg/day within 7 days prior to the first Intuvax dose. Inhaled, intranasal and local steroids accepted (irrespective of dose)
- Inadequate laboratory parameters (i.e. B-leukocyte count < 3.0 x 10^9^/L; B-platelet count < 75 x 10^9^/L; B-hemoglobin <100 g/L; P-prothrombincomplex >1.4; P-APT time outside of normal limit)
- Previous organ transplantation
- Pregnant/lactating women
- Life expectancy less than 3 months
- Investigational treatment (within 28 days) prior to the first injection of Intuvax
- Known blood dyscrasia (bleeding complication)
- Prior history of invasive cancer within 5 years before screening (except for adequately treated *in situ* carcinomas or non-melanoma skin cancer)
- History of alcohol/substance abuse
- Patient will not be available for follow-up assessments

Any other reason that, in the opinion of the investigator, contraindicates that the subject participates in the trial
